# Outcomes of Patients with Preoperative Thrombocytosis After Hip Fracture Surgery

**DOI:** 10.5435/JAAOSGlobal-D-23-00159

**Published:** 2024-04-09

**Authors:** Christian A. Gonzalez, Noelle L. Van Rysselberghe, Clayton Maschhoff, Michael J. Gardner

**Affiliations:** From the Reno School of Medicine, University of Nevada, Reno, NV (Dr. Gonzalez); the Department of Orthopaedic Surgery, Stanford University, Stanford, CA (Dr. Rysselberghe and Dr. Gardner); and the University of Illinois College of Medicine, Chicago, IL (Dr. Maschhoff).

## Abstract

**Introduction:**

Low platelet counts have clinically relevant effects on patient outcomes after hip fracture surgery; however, the relationship between abnormally high platelet counts and postoperative outcomes in this population is unknown.

**Methods:**

The ACS-NSQIP database was queried for patients who underwent hip fracture surgery between 2015 and 2019. Outcomes were compared between patients with normal platelet counts (150,000 to 450,000/μL) and thrombocytosis (>450,000/μL).

**Results:**

Eighty-six thousand three hundred eleven hip fracture patients were identified, of which 1067 (1.2%) had preoperative thrombocytosis. Compared with patients with normal platelet counts, patients with preoperative thrombocytosis had increased rates of 30-day mortality (6.4% vs 4.5%, *P* = 0.004; OR 1.15 [95% CI 0.88 to 1.50], *P* = 0.322) as well as increased rates and odds of readmission (11.4% vs 7.8%, *P* < 0.001; OR 1.35 [95% CI 1.10 to 1.65], *P* = 0.004) and venous thromboembolic events (3.2% vs 1.7%, *P* < 0.001; OR 1.88 [95% CI 1.31 to 2.71], *P* < 0.001).

**Conclusions:**

Hip fracture patients with preoperative thrombocytosis had increased rates of early mortality as well as increased odds of venous thromboembolic events and readmission. A patient with thrombocytosis may benefit from close postoperative surveillance and careful follow-up. Future prospective studies are needed to verify causation and investigate how to mitigate adverse outcomes in hip fracture patients with preoperative thrombocytosis.

Hip fractures result in high morbidity, mortality, and economic burden. One-year mortality rates have been reported in up to 20 to 30% patients, and many surviving patients experience notable functional loss.^1-5^ As the population ages, the incidence of hip fractures in the United States is projected to increase and cost over $18 billion by 2025.^1,2,5^

Elevated platelet counts have clinically relevant effects on patient outcomes in cardiac, general, neurologic, oncologic, and vascular surgery.^6-10^ Recently, abnormally high platelet counts were associated with major and minor adverse postoperative events in shoulder and total hip arthroplasties and minor adverse postoperative events in total knee arthroplasty.^11-13^ A recent retrospective review of 72,306 hip fracture patients found that preoperative platelet counts <150,000/μL were associated with an increase in early mortality, which was especially pronounced in patients with platelet counts between 50,000/μL and 100,000/μL (OR 1.63 [95% CI 1.41 to 1.90], *P* < 0.001).^14^ However, the authors grouped elevated platelet counts with normal platelet counts.^14^ The relationship between abnormally high platelet counts and postoperative outcomes in the hip fracture population is unknown.

The purpose of this study was to use a large, nationally representative database to determine the incidence of preoperative thrombocytosis and the associations with 30-day postoperative infections, venous thromboembolic events (VTEs), mortality, readmission, and unplanned revision surgery in patients undergoing hip fracture surgery. We hypothesized that preoperative thrombocytosis (defined as platelet count >450,000/μL) would be associated with increased adverse postoperative outcomes in this patient population.

## Methods

The American College of Surgeons (ACS) National Surgical Quality Improvement Program (NSQIP) Participant Use Data File was used for this investigation. The ACS NSQIP contains deidentified patient information, surgical details, and 30-day postoperative outcomes.^15^ Data are obtained from diverse healthcare settings across the United States, and in 2019, the Participant Use Data File contained 1,076,441 cases submitted from 719 locations.^15,16^ Data are prospectively collected by a trained surgical clinical reviewer, and previous studies have demonstrated a 98% inter-rater agreement rate between reviewers and excellent reliability.^15-17^ This research is HIPAA-compliant and institutional review board–exempt.

Orthopaedic surgery cases from the 2015 to 2019 ACS NSQIP were initially included. Current Procedural Terminology (CPT) codes representative of hip fractures including hemiarthroplasty (27,236), open reduction and internal fixation (27,244), and intramedullary nailing (27,245) were used to identify patients.^16^

Patient characteristics collected from the registry included age, sex, weight, height, and comorbidities. Comorbidities included American Society of Anesthesiologists (ASA) class, diabetes (DM), hypertension (HTN), congestive heart failure (CHF), smoking, chronic obstructive pulmonary disease (COPD), disseminated cancer, dialysis, and functional status. Body mass index was calculated from each patient's height and weight. Age was grouped 18 to 55, 56 to 70, 71 to 85, and greater than 85 years.

A small proportion of patients with missing ASA class, weight, height, or functional status were excluded before analysis. Preoperative hematocrit <36% for women or <39% for men was used to define anemia.^18,19^ Preoperative white blood cell counts were additionally collected as a potential confounder for adverse outcomes and classified as leukocytosis for WBC counts greater than 11,000/μL, leukopenia for WBC counts less than 4,000/μL, and normal WBC count 4000 to 11,000/μL. All hip fracture patients with available preoperative platelet counts were initially included to report the incidence of preoperative thrombocytosis. The remaining patients were placed into three groups based on their preoperative platelet counts as follows: (1) less than 150,000/μL (thrombocytopenia), (2) between 150,000 and 450,000/μL (normal), and (3) greater than 450,000/μL (thrombocytosis).^20^ Patients with thrombocytopenia and patients with preoperative infections were excluded for the analysis.

Analyzed outcomes included mortality, unplanned return to the operating room, any readmission, postoperative venous thromboembolism (deep vein thrombosis or pulmonary embolism), or infectious complications (sepsis, pneumonia, or surgical site infection [which included superficial, deep, or organ site infections]) within 30 days of surgery.

## Statistical Analysis

Descriptive statistics were used to summarize baseline characteristics. Chi square test was used to analyze baseline differences and initial differences in outcomes between patients with preoperative thrombocytosis and patients with normal preoperative platelet counts. A multivariable regression model which was controlled for age, sex, BMI, ASA class, type of surgery, diabetes, HTN, CHF, smoking, COPD, disseminated cancer, dialysis, anemia, and leukocytosis was used to compare postoperative outcomes between patients with preoperative thrombocytosis and patients with normal preoperative platelet counts. Collinearity was assessed by using a variance inflation factor (VIF) analysis. Variables were considered collinear if the VIF was greater than 2.5. Significance was defined as *P* values less than 0.05. All statistical analyses were conducted using IBM SPSS v.28.0 (SPSS IBM).

## Results

A total of 86,311 hip fractures patients were initially included, of which 1067 (1.2%) had preoperative thrombocytosis and 17,668 (20.5%) had preoperative thrombocytopenia. After patients with thrombocytopenia or preoperative infections were excluded, there were 65,875 hip fracture patients. Overall, 985 patients (1.5%) had preoperative thrombocytosis and 64,890 patients (98.5%) had normal preoperative platelet counts for the control group.

Compared with patients with normal platelet counts, patients with preoperative thrombocytosis were markedly more likely to be younger, female, actively smoking, and have COPD or disseminated cancer. The mean ASA score in patients with thrombocytosis was significantly higher (3.1 vs 3.0, *P* < 0.001). The proportion of thrombocytosis patients with obesity was markedly lower compared with those with normal platelet counts. Diabetes, HTN, CHF, and dialysis were not markedly different between the groups. After excluding patients with preoperative infections, significantly more patients with thrombocytosis had concurrent leukocytosis (Table 1). The rates of preoperative thrombocytosis among hip fracture patients who were treated with hemiarthroplasty (1.6%), ORIF (1.4%), or an intramedullary nail (1.4%) were not different (*P* = 0.058).

**Table 1:** Patient Characteristics and Comorbidities by Preoperative Thrombocytosis Status

	Thrombocytosis	Normal Platelet Count	*P*
Age (yr)			<0.001
18-55	72 (7.3%)	2626 (4.0%)	
56-70	218 (22.1%)	10,839 (16.7%)	
71-85	400 (40.6%)	27,705 (42.7%)	
> 85	295 (29.9%)	23,720 (36.6%)	
BMI			<0.001
≤ 25	664 (67.4%)	35,940 (55.6%)	
>25-30	211 (21.4%)	18,259 (28.0%)	
>30	110 (11.2%)	10,691 (16.4%)	
Female	747 (75.8%)	47,025 (72.5%)	0.019
Diabetes	162 (16.4%)	11,849 (18.2%)	0.143
Hypertension	624 (63.4%)	42,308 (65.2%)	0.227
Smoking	208 (21.1%)	8409 (13.0%)	<0.001
COPD	179 (18.2%)	7092 (10.9%)	<0.001
CHF	37 (3.8%)	1965 (3.0%)	0.186
Disseminated cancer	105 (10.7%)	1889 (2.9%)	<0.001
Dialysis	7 (0.7%)	934 (1.4%)	0.056
Mean ASA	3.1	3.0	<0.001
Leukocytosis	360 (36.5%)	8204 (12.6%)	<0.001

COPD = chronic obstructive pulmonary disease, CHF = congestive heart failure

A bivariate analysis was conducted to assess outcomes and the association with preoperative thrombocytosis in all patients (Table 2) and within subgroups of each type of surgery (Supplemental Tables 1-3, http://links.lww.com/JG9/A333). Compared with patients with normal platelets counts, patients with preoperative thrombocytosis had increased rates of 30-day mortality (6.4% vs 4.5%, *P* = 0.004), unplanned revision surgery (4.1% vs 2.2%, *P* < 0.001), readmission (11.4% vs 7.8%, *P* < 0.001), VTEs (3.2% vs 1.7%, *P* < 0.001), any infection (8.5% vs 5.0%, *P* < 0.001), SSIs (2.0% vs 1.0%, *P* = 0.002), pneumonia (5.5% vs 3.5%, *P* < 0.001), and sepsis (1.7% vs 0.9%, *P* = 0.007).

**Table 2:** Bivariate Analysis of Outcomes by Preoperative Thrombocytosis Status

	Thrombocytosis	Normal Platelet Count	*P*
30-Day mortality	63 (6.4%)	2917 (4.5%)	0.004
Unplanned revision surgery	40 (4.1%)	1430 (2.2%)	<0.001
Readmission	112 (11.4%)	5067 (7.8%)	<0.001
VTE	32 (3.2%)	1095 (1.7%)	<0.001
Any infection	84 (8.5%)	3252 (5.0%)	<0.001
SSI	20 (2.0%)	670 (1.0%)	0.002
Pneumonia	54 (5.5%)	2249 (3.5%)	<0.001
Sepsis	17 (1.7%)	585 (0.9%)	0.007

VTE = venous thromboembolic event, SSI = surgical site infection.

A multivariable analysis that was controlled for all patient characteristics and comorbidities was conducted to test whether preoperative thrombocytosis independently predicted adverse postoperative events (Table 3). This multivariable analysis revealed that, compared with patients with normal platelets counts, patients with preoperative thrombocytosis had significantly increased odds of unplanned revision surgery (OR 1.72 [95% CI 1.24 to 2.38], *P* < 0.001), readmission (OR 1.35 [95% CI 1.10 to 1.65], *P* = 0.004), VTE (OR 1.88 [95% CI 1.31 to 2.71], *P* < 0.001), any infection (OR 1.42 [95% CI 1.13 to 1.79], *P* = 0.003), and SSI (OR 1.91 [95% CI 1.21 to 3.02], *P* = 0.005). Preoperative thrombocytosis did not independently predict higher odds of 30-day mortality, pneumonia, or sepsis. No problem was found with data collinearity (tolerance was not less than 0.1 and the VIF was < 2.5 for all measured variables).

**Table 3:** Multivariable Analysis of Outcomes by Preoperative Thrombocytosis Status

	Odds Ratio (95% CI)	*P*
30-Day mortality	1.15 (0.88-1.50)	0.322
Unplanned Revision surgery	1.72 (1.24-2.38)	<0.001
Readmission	1.35 (1.10-1.65)	0.004
VTE	1.88 (1.31-2.71)	<0.001
Any infection	1.42 (1.13-1.79)	0.003
SSI	1.91 (1.21-3.02)	0.005
Pneumonia	1.23 (0.93-1.64)	0.150
Sepsis	1.59 (0.97-2.60)	0.067

CI = confidence interval, VTE = venous thromboembolic event, SSI = surgical site infection.

## Discussion

As the population ages and the incidence of hip fractures increases, it is prudent to understand preoperative markers that predispose patients to adverse postoperative events.^1^ The incidence of thrombocytosis in this surgical hip fracture population was 1.2%, which is within the 0.4 to 1.7% range of rates in arthroplasty patients.^11-13^ This study found that preoperative thrombocytosis was associated with increased rates of 30-day mortality, unplanned revision surgery, readmission, VTE, and infections after hip fracture surgery.

While the etiology of thrombocytosis was unknown, reactive thrombocytosis is the leading cause of thrombocytosis in the general medical population and usually occurs as a result of an acute phase response to infection or tissue damage.^7,21,22^ Prior studies found that infected patients may present with thrombocytosis even in the absence of other more obvious signs of infection such as an elevated white blood cell count or fever.^23,24^ In agreement with the literature in other patient populations,^8-10^ our findings suggest that thrombocytosis may be a clinical indicator of postoperative infection risk in hip fracture patients. After excluding all patients with preoperative infections and controlling for patient characteristics and comorbidities including white blood cell count, preoperative thrombocytosis was associated with increased odds of having any postoperative infection (OR 1.42 [95% CI 1.13 to 1.79], *P* = 0.003).

Thrombocytosis in the critically ill or in the setting of tissue damage after injury has been reported to put patients at increased risk of thromboembolic events.^7,25-28^ Similarly, hip fracture patients with preoperative thrombocytosis in this study had increased odds of VTE compared with patients with normal platelet counts (OR 1.88 [95% CI 1.31 to 2.71], *P* < 0.001). It has been postulated that reactive thrombocytosis may be a surrogate marker of a prothrombotic state, likely as a consequence of platelet hyperactivity, with some authors arguing in favor of a causal relationship between thrombocytosis and VTE.^26-30^

This study did not address the effects of preoperative thrombocytopenia (platelets <150,000/μL) in hip fracture patients, which has previously been associated with increased postoperative bleeding events and early mortality compared with patients with platelet counts >150,000/μL.^14^ Taking these findings together, it seems likely that both thrombocytopenia and thrombocytosis have negative postoperative consequences, with the former being predisposed to bleeding events and the latter to VTE events and infection. In addition, because previous studies have grouped normal platelet counts with thrombocytosis,^14^ it is possible that the effect of low preoperative platelet counts on early mortality may be even more pronounced. To our knowledge, this is the first study to examine adverse postoperative events associated with elevated preoperative platelet counts in orthopaedic trauma surgery. Based on these findings, we encourage physicians to be cognizant of the risks associated with not only low preoperative platelet counts but also high preoperative platelet counts in patients undergoing hip fracture surgery.

The time-sensitive nature of hip fractures constrains preoperative optimization, and evidence to guide management for patients with thrombocytosis is lacking. Patients with thrombocytosis may benefit from close postoperative surveillance, consideration of possible concomitant infectious conditions, and careful follow-up. This study found preoperative thrombocytosis to be associated with a 1.5% absolute increase in the rate of VTE and was an independent risk factor after multivariable adjustment. VTE chemoprophylaxis is the standard of care after hip fracture surgery, and physicians must cautiously weigh the risks and benefits of initiating VTE chemoprophylaxis in patients with relative contraindications.^31,32^ Future prospective studies are needed to verify causation and investigate how to mitigate adverse outcomes in hip fracture patients with preoperative thrombocytosis.

This study had the expected limitations of retrospective research with a large database. However, the standardized collection process and validation of the NSQIP mitigate some of the recording as well as measurement bias that goes along with this type of research. Only including data within 30 days of surgery may falsely lower the number of adverse events. Some patients were excluded for missing data. Although this number was small (<3%), it still introduces some variability in the reported incidence of thrombocytosis. Baseline differences were noted between the groups, and the potential for confounding variables may limit the results of our bivariate analysis. This limitation was mitigated by controlling for these differences and other patient characteristics and comorbidities in the multivariable analyses. In addition, we assessed for data collinearity and reported this was not a problem. There may have been infections not specifically recorded in the NSQIP that were included, and it is inevitable that patients with unidentified infections were included. However, this was controlled to the best of our abilities within the confines of the NSQIP database by excluding patients with known preoperative infections and controlling for preoperative white blood cell count in the analyses. Preoperative patient temperatures, injury severity, perioperative interventions targeted at a patient's platelet count, and VTE prophylaxis are not recorded in the NSQIP and, therefore, were unable to be assessed or controlled. Controlling for other comorbidities in our analyses helped mitigate these limitations.

## Conclusion

This research provides the initial report of the risks associated with preoperative thrombocytosis in the orthopaedic trauma literature. The incidence of thrombocytosis in the surgical hip fracture population was 1.2% and was associated with increased rates of mortality compared with patients with normal platelet counts. Our findings suggest that hip fracture patients with preoperative thrombocytosis have an increased risk of readmission, unplanned revision surgery, VTE, and postoperative infections after controlling for patient characteristics and comorbidities. A patient with thrombocytosis may benefit from close postoperative surveillance, consideration of possible concomitant infectious conditions, and careful follow-up. Future prospective studies are needed to verify causation and investigate how to mitigate adverse outcomes in hip fracture patients with preoperative thrombocytosis.

## Supplementary Material
