# Supplementary material for: Outcomes of Patients with Preoperative Thrombocytosis After Hip Fracture Surgery
Source: J Am Acad Orthop Surg Glob Res Rev. 2024 Apr 9;8(4):e23.00159. doi: 10.5435/JAAOSGlobal-D-23-00159 (PMC11003512; doi:10.5435/JAAOSGlobal-D-23-00159)
Supplement: Supplementary file 1 [file jagrr-8-e23.00159-s001.docx]

Supplemental Table 1: Bivariate Analysis of Outcomes by Preoperative Thrombocytosis Status in Hemiarthroplasty Patients

|  | Thrombocytosis | Normal Platelet Count | p value |
| --- | --- | --- | --- |
| 30 Day Mortality | 27 (7.0%) | 992 (4.3%) | 0.010 |
| Unplanned Reoperation | 18 (4.7%) | 674 (2.9%) | 0.045 |
| Readmission | 54 (14.0%) | 1903 (8.3%) | <0.001 |
| VTE | 15 (3.9%) | 358 (1.6%) | <0.001 |
| Any Infection | 34 (8.8%) | 1226 (5.3%) | 0.003 |
| SSI | 12 (3.1%) | 313 (1.4%) | 0.004 |
| Pneumonia | 20 (5.2%) | 803 (3.5%) | 0.072 |
| Sepsis | 4 (1.0%) | 211 (0.9%) | 0.804 |

VTE, venous thromboembolic events; SSI, surgical site infection.

Supplemental Table 2: Bivariate Analysis of Outcomes by Preoperative Thrombocytosis Status in ORIF Patients

|  | Thrombocytosis | Normal Platelet Count | p value |
| --- | --- | --- | --- |
| 30 Day Mortality | 7 (7.1%) | 283 (4.0%) | 0.122 |
| Unplanned Reoperation | 3 (3.0%) | 136 (1.9%) | 0.424 |
| Readmission | 8 (8.1%) | 479 (6.8%) | 0.602 |
| VTE | 1 (1.0%) | 100 (1.4%) | 0.737 |
| Any Infection | 18 (18.2%) | 341 (4.8%) | <0.001 |
| SSI | 2 (2.0%) | 62 (0.9%) | 0.228 |
| Pneumonia | 11 (11.1%) | 249 (3.5%) | <0.001 |
| Sepsis | 8 (8.1%) | 54 (0.8%) | <0.001 |

ORIF, open reduction and internal fixation; VTE, venous thromboembolic events; SSI, surgical site infection.

Supplemental Table 3: Bivariate Analysis of Outcomes by Preoperative Thrombocytosis Status in IMN Patients

|  | Thrombocytosis | Normal Platelet Count | p value |
| --- | --- | --- | --- |
| 30 Day Mortality | 29 (5.8%) | 1642 (4.7%) | 0.263 |
| Unplanned Reoperation | 19 (3.8%) | 620 (1.8%) | <0.001 |
| Readmission | 50 (10.0%) | 2685 (7.7%) | 0.060 |
| VTE | 16 (3.2%) | 637 (1.8%) | 0.025 |
| Any Infection | 32 (6.4%) | 1685 (4.8%) | 0.110 |
| SSI | 6 (1.2%) | 295 (0.8%) | 0.398 |
| Pneumonia | 23 (4.6%) | 1197 (3.4%) | 0.161 |
| Sepsis | 5 (1.0%) | 320 (0.9%) | 0.855 |

IMN, intramedullary nail; VTE, venous thromboembolic events; SSI, surgical site infection.
